# Tuning excitatory input to fast-spiking parvalbumin-positive interneurons: a lever for plasticity and hyperexcitability across the lifespan

**DOI:** 10.3389/fnsyn.2026.1819500

**Published:** 2026-04-24

**Authors:** Daniel Severin, Alfredo Kirkwood

**Affiliations:** 1 Mind/Brain Institute, Johns Hopkins University, Baltimore, MD, United States; 2 Department of Neurosciences, Johns Hopkins University, Baltimore, MD, United States

**Keywords:** age related cognitive decline, Alzheimer’s disease, critical period, excitatory inputs, fast-spiking (FS) parvalbumin (PV)-interneurons, hippocampal hyperexcitability, NPTX2, ocular dominance plasticity

## Abstract

Experience reshapes cortical circuits, yet plasticity is tightly gated—high during early critical periods and increasingly constrained with maturation. Later in life, aging and Alzheimer’s disease (AD) create a growing demand to restrain network hyperactivity. Across these contexts, excitatory drive onto parvalbumin-positive fast-spiking interneurons (PVs)—shaped by synaptic organizers such as NPTX2—offers a control point for tuning inhibitory tone while preserving fast, precise inhibition. We outline the cellular and synaptic specializations that make PVs powerful regulators of network excitability, then synthesize evidence from visual cortex suggesting that critical period termination reflects the loss of plasticity at principal neuron→PV inputs. Finally, we extend this framework to aging and AD, where medial temporal lobe hyperactivity and early PV dysfunction coincide with NPTX2 dysregulation, suggesting that restoring excitatory recruitment of PVs may help stabilize circuits and prevent cognitive decline.

## Introduction

Parvalbumin-positive fast-spiking interneurons (PVs) are the most abundant class of cortical inhibitory interneurons (Rudy et al., 2011); here we focus on basket cells. PVs sustain high-frequency firing with little or no adaptation and impose precise spatiotemporal control over neuronal activity (reviewed in Hu et al., 2014; Tremblay et al., 2016; Bartholome et al., 2020). They are therefore central to network oscillations (Bartos et al., 2007; Stark et al., 2013, 2014), excitation–inhibition balance (Hasenstaub et al., 2005; Haider and McCormick, 2009; Sadeh and Clopath, 2021), cortical gain control (Roux and Buzsáki, 2015), and the regulation of plasticity and learning (Yazaki-Sugiyama et al., 2009; Letzkus et al., 2011; Donato et al., 2013; Kuhlman et al., 2013). At the microcircuit level, PVs are tightly coupled to excitatory principal neurons (PNs) and provide both feedforward and feedback inhibition, enabled by a phenotype optimized for rapid transmission (Hu et al., 2014; Tremblay et al., 2016). This speed and precision arise from cellular and synaptic specializations that minimize signaling delay and temporal jitter. While PVs share these core features across brain regions (Kepecs and Fishell, 2014), we focus here on hippocampus and neocortex.

These specializations span the entire signaling chain, from postsynaptic receptors to axon terminals. At excitatory synapses onto PVs, postsynaptic receptors are optimized for speed (Hu et al., 2014; Tremblay et al., 2016), and PV membranes are tuned for rapid voltage responses: low membrane resistance and capacitance shorten the time constant, limit temporal summation, and favor rapid activation (Milicevic et al., 2024). High dendritic K^+^/Na^+^ conductance further accelerates repolarization and supports fast spiking (Hu et al., 2010; Rudy and McBain, 2001). Kv3 channels are especially prominent in PVs and play a central role: because they activate and deactivate rapidly at relatively depolarized voltages, they accelerate action-potential repolarization and facilitate recovery from Na^+^ channel inactivation, enabling sustained high-frequency firing (Erisir et al., 1999; Rudy and McBain, 2001; Kaczmarek and Zhang, 2017). At axon terminals, Kv3 channels sharpen presynaptic spikes and preserve Ca^2+^ channel availability during high-frequency firing, while P/Q-type Ca^2+^ channels—with their fast kinetics and nanodomain coupling—support rapid and reliable transmitter release (Goldberg et al., 2005; Tremblay et al., 2016). Proximal axon myelination and supercritical Na^+^ channel density further accelerate conduction (Stedehouder et al., 2017; Hu and Jonas, 2014). Beyond these structural features, PV synapses can recover from short-term depression in an activity-dependent manner by adjusting vesicle replenishment, allowing PVs to suppress PN firing across a broad frequency range and stabilize network activity (Bridi et al., 2020).

The specializations described above raise the question of how PV recruitment can be adjusted without compromising rapid, temporally precise inhibition. Although activity-dependent regulation is well characterized for principal neurons, how activity modulates PV circuits is less clear, though several forms of PV plasticity are beginning to emerge (McFarlan et al., 2023). These include changes in intrinsic excitability (Donato et al., 2013; Dehorter et al., 2015; Tzilivaki et al., 2023), PV inhibitory output, and excitatory input. PV output in adult cortex is often stable (Morales et al., 2002; Chattopadhyaya et al., 2004; Jiang et al., 2010; Gao et al., 2017), although forms of output plasticity have been reported, with mechanisms that are only beginning to be understood (Capogna et al., 2021; Hadler et al., 2024). Recent examples include peptide-dependent regulation of PV output: Vgf influences structural PV → PV connectivity (Selten et al., 2025), and Scg2-derived peptides potentiate GABAergic transmission (Yap et al., 2021). While intrinsic and output plasticity may serve important roles, these properties are tightly linked to the features that confer rapid, precise inhibition, and modulating them may constrain PV function. By contrast, excitatory input plasticity operates upstream of these specializations, providing a parsimonious mechanism for adjusting inhibitory tone without altering the defining transmission properties through which PVs shape network dynamics (Gao et al., 2017). Indeed, excitatory inputs onto PVs undergo activity-dependent synaptic plasticity, including Hebbian and anti-Hebbian forms (Lu et al., 2007; Huang et al., 2013; Chou et al., 2025), as well as rapid, experience-dependent change (Kuhlman et al., 2013).

PV interneurons are ensheathed by perineuronal nets (PNNs)—activity-regulated extracellular matrix structures that regulate AMPA receptor availability at excitatory synapses onto PVs, making them a well-established locus for tuning PV recruitment (Favuzzi et al., 2017). Among the molecular organizers operating within this environment, NPTX2 (NARP/NP2) stands out because it is activity regulated, enriched at excitatory synapses onto PVs, and mechanistically well characterized (Okur and Scheiffele, 2025). NPTX2 is expressed by excitatory neurons and secreted to form pentraxin complexes with NP1 and NPR that stabilize and cluster AMPA receptors at PV inputs (Chang et al., 2010; Pelkey et al., 2015). It also promotes PNN formation, supports synapse maturation, and sustains PV excitation across life; in visual cortex, NPTX2 contributes to excitatory connectivity and ocular dominance plasticity (Gu et al., 2013; Van’t Spijker et al., 2019).

These properties position NPTX2 to couple ongoing principal-neuron activity to rapid remodeling of PV recruitment. Synaptic NPTX2 levels fluctuate within minutes of evoked activity, and both NPTX2 and excitatory synapses onto PVs oscillate across the 24-h day (Xiao et al., 2021; Zong et al., 2023), indicating that this system is dynamically and bidirectionally regulated. This rapid bidirectional regulation makes NPTX2-stabilized PN → PV connectivity well suited for homeostatic negative feedback, in which fluctuations in PN activity are countered by compensatory changes in PV recruitment to stabilize circuit gain (Chang et al., 2010). Beyond graded adjustments, NPTX2-dependent mechanisms may also support all-or-none synapse elimination. The rapid disconnection of PN → PV connections observed during ODP suggests a form of plasticity involving elimination rather than weakening alone (Severin et al., 2021). Consistent with this idea, associative LTD at PN → PV synapses and synapse elimination in CA1 PNs both require mGluR5 activation, raising the possibility that graded weakening and complete disconnection share common upstream signaling (Huang et al., 2013; Wilkerson et al., 2014). At the extracellular level, NPTX2 binds complement factor C1q and limits microglial activation (Zhou et al., 2023), suggesting that NPTX2 shedding permits C1q-mediated microglial engulfment and synapse removal. These findings position NPTX2 as a versatile regulator of PN → PV connectivity, potentially contributing to both rapid homeostatic tuning and structural loss of connectivity under conditions of circuit remodeling.

This evidence suggests that PV interneurons—despite their highly specialized fast inhibitory output—are regulated upstream by activity-dependent tuning of their excitatory drive, with NPTX2 well positioned as a synaptic organizer of this process. To examine this idea across the lifespan, we focus on two well-established models. The first is ocular dominance plasticity (ODP) in visual cortex, a canonical system for studying experience-dependent juvenile circuit refinement. Here, we synthesize work showing that diverse interventions reinstating ODP in adults converge on remodeling PV circuitry, and we propose that opening and closing the critical period are governed by the maturation of excitatory inputs onto PVs and the subsequent loss of plasticity at those inputs. The second is age-related dysfunction in medial temporal lobe (MTL)^1^ circuits in aging and Alzheimer’s disease (AD), where hyperactivity^2^ and PV dysfunction are linked to memory impairment. Here, we explore how re-tuning PV recruitment may help constrain MTL hyperactivity and support cognitive performance. Comparing these models allows us to ask whether regulating PV recruitment through excitatory inputs serves as a common circuit lever that permits plasticity in youth while restraining hyperexcitability^3^ in aging. In the Discussion, we evaluate the strengths and limits of this perspective and outline key open questions.

## Excitatory drive onto PVs gates the ocular dominance critical period

A permissive range of inhibitory tone has been proposed as necessary for the expression of the ODP critical period (for review, see Jiang et al., 2005; Sale et al., 2010; Levelt and Hübener, 2012; Hensch and Quinlan, 2018). While maturation of PV-mediated inhibition provides a well-established trigger for critical period onset, what drives its termination is less clear. Numerous interventions enable or enhance ODP in adult animals, and although some act through pathways beyond inhibition, a predominant pattern involves disinhibition via altered inhibitory circuits (for reviews, see Levelt and Hübener, 2012; Trachtenberg, 2015; Reh et al., 2020; Qin et al., 2025). Yet ODP persists even after the developmental maturation of perisomatic inhibition (Sawtell et al., 2003; Pham et al., 2004; Lehmann and Löwel, 2008; Huang et al., 2010), suggesting that key regulatory control resides at the level of excitatory drive onto PVs rather than PV output. In this context, we proposed that maturation of excitatory drive onto PV interneurons determines the timing of the critical period (Huang et al., 2010; Gu et al., 2013).

Consistent with a recruitment-based mechanism, one day after monocular deprivation (MD), visually evoked responses in L2/3 PNs are 2 × stronger than in controls (Kuhlman et al., 2013; Sun et al., 2016). This increase reflects a rapid reduction in excitatory drive onto L2/3 PVs, eliminating 50% of their local excitatory inputs and producing a transient disinhibition that enables subsequent ODP (Kuhlman et al., 2013; Sun et al., 2016; Severin et al., 2021). While other mechanisms may contribute, reduced PV recruitment appears to be the primary driver of this disinhibitory shift.

What molecular mechanisms underlie this reduction in PV recruitment? Several proteins—including SynCAM1, Nogo receptor 1 (NgR1), neuregulin 1 (NRG1), and NPTX2—localize to excitatory synapses onto PVs, and disrupting them blunts the disinhibition found after 1 day of MD (Stephany et al., 2014; Ribic et al., 2019; Sun et al., 2016; Gu et al., 2016; Severin et al., 2021). These molecules tune distinct sources of excitation: in L2/3, NPTX2 regulates local intralaminar drive, whereas in L4, NgR1 influences intracortical inputs and SynCAM1 modulates thalamocortical excitation (Frantz et al., 2020; Ribic et al., 2019). NRG1 acts on L4-derived pathways and may gate ODP locally within L4, consistent with the finding that L4 → L2/3 synapses onto PVs are unchanged after 1 day of MD (Severin et al., 2021). Exogenous NRG1 rescues the NPTX2 knockout phenotype, indicating that these pathways independently regulate excitation onto PVs (Gu et al., 2016). Because NPTX2 regulates the local inputs that are remodeled during MD, it is well positioned to govern critical period timing.

Which regulator dominates likely depends on circuit context and developmental stage. Our ODP work shows that manipulating NPTX2 to block or promote the transient disconnection of PN → PV inputs correspondingly prevents or enables ocular dominance shifts after MD, suggesting that critical-period closure reflects a loss of plasticity at PN → PV synapses (Severin et al., 2021). In this framework, NPTX2 serves as a permissive organizer of PN → PV plasticity, allowing the appropriate neuronal activity regime to remodel these synapses—a role that, as we discuss next, extends beyond developmental plasticity to age-related circuit dysfunction.

## PV recruitment constrains hyperexcitability in aging and Alzheimer’s disease

We next ask whether the same PV-recruitment framework that governs the critical period in visual cortex also operates in MTL circuits during aging, where a central demand is to restrain hyperexcitability and preserve memory. Across species and disease contexts, neuronal hyperactivity in the MTL is a recurring correlate of cognitive impairment in aged rats (Wilson et al., 2005), Alzheimer’s disease mouse models (Palop et al., 2007), and older human adults with mild cognitive impairment (MCI) (Yassa et al., 2011). However, the molecular mechanisms driving this hyperactivity remain incompletely understood. The PV-recruitment model offers an explanation: the same synaptic locus—excitatory drive onto PVs—that is adaptively tuned during the critical period becomes a point of vulnerability in aging when this regulation fails, potentially permitting the hyperexcitability observed in these conditions.

The detrimental effects of excessive neural activity on memory during aging underscore the importance of maintaining a dynamic balance between synaptic excitation and inhibition. This balance is critical for the synaptic plasticity mechanisms that underlie memory encoding (Keck et al., 2017; Denève et al., 2017). It helps set postsynaptic depolarization and the resulting Ca^2+^ influx that bias whether patterned activity induces LTP or LTD, while preventing self-reinforcing, uncontrolled changes in synaptic strength (Steele and Mauk, 1999; Cummings et al., 1996). Consistent with this mechanism, circadian shifts in hippocampal E/I balance can alter CA1 long-term plasticity (Valdivia et al., 2026).

PV interneurons, as the dominant source of perisomatic inhibition, are central to maintaining E/I balance. Excitatory drive onto them directly supports memory function: fast potentiation of input from dentate gyrus granule cells onto PVs in CA3 is crucial for the consolidation and maintenance of new memories (Guo et al., 2018). Despite this critical role, PV function is disrupted in both AD and normal aging, with alterations converging on early PV changes linked to neuronal hyperactivity (Palop and Mucke, 2016; Černotová et al., 2023; Hijazi et al., 2023; Zhang et al., 2025; Hadler et al., 2024). Age- or AD-related declines in NPTX2 may progressively weaken excitatory drive onto MTL PVs, reducing their recruitment and permitting the hyperexcitability observed in these conditions. These findings suggest that modulating PV-dependent network alterations could be a useful therapeutic strategy to improve brain function in aging and AD (Palop and Mucke, 2016; Hijazi et al., 2023; Zhang et al., 2025; Hadler et al., 2024).

Among potential molecular targets, NPTX2 is particularly compelling in the context of age-dependent PV dysfunction: it is highly expressed in MTL structures, and reduced cerebrospinal fluid NPTX2 tracks with poorer cognitive performance (Swanson et al., 2016; Xiao et al., 2017; Libiger et al., 2021). Reduced NPTX2 also predicts progression from normal cognition to MCI (Soldan et al., 2023), consistent with diminished recruitment of PV interneurons, as suggested by NPTX2−/− mice (Gu et al., 2013). Recent studies provide direct experimental support. In the visual cortex of AD mouse models, layer 5 neurons show abnormally prolonged Ca^2+^ transients before substantial plaque formation, and NPTX2 overexpression increases excitatory input onto layer 5/6 PVs and restores their spiking activity (Papanikolaou et al., 2025). In aged learning-impaired rats, viral upregulation of NPTX2 in lateral entorhinal cortex → dentate gyrus projections increases excitatory input onto PVs, improves cognitive performance, and restores E/I balance in the dentate gyrus (Severin et al., 2025). These manipulations directly test the hypothesis that reduced PV recruitment contributes to age-related dysfunction: restoring NPTX2 restores PV recruitment, and restoring PV recruitment restores function.

Taken together, the evidence indicates that aging and AD feature MTL hyperactivity alongside early, NPTX2-sensitive alterations in PV recruitment. This extends the PV-recruitment framework from developmental plasticity to late-life circuit dysfunction, positioning PV recruitment as a central, testable lever for restoring E/I balance in aging and AD.

## Discussion

The evidence reviewed above supports a unifying perspective: Recruiting PV interneurons through their excitatory inputs offers a way to tune inhibitory tone upstream—without perturbing the coordinated physiological and molecular specializations that enable PVs’ fast inhibitory transmission. PV recruitment serves distinct roles across life stages and operates over timescales from minutes to long-lasting changes. It is also bidirectionally regulated to adjust inhibitory tone contingent on circuit demand. Among the mechanisms that shape PV recruitment, NPTX2 is a particularly compelling node because it provides a direct, activity-coupled handle on PV excitation and has a well-defined molecular pathway.

Building on this idea, we posit PV recruitment as a key control point for circuit excitability, implemented by a multi-dial system in which distinct molecular pathways tune PV recruitment. The range of manipulations that shift plasticity or excitability suggests many knobs exist, but the brain is unlikely to engage them all at once; instead, the dominant dial likely depends on local circuit architecture, developmental stage, and behavioral demand. This also implies a limitation of the present focus: while we have emphasized NPTX2 because of its well-characterized pathway and activity dependence, NPTX2 likely operates as the dominant regulator only in specific contexts, and other molecular programs may govern PV recruitment elsewhere. For example, in visual cortex, NPTX2 appears anatomically and functionally segregated from other pathways: it is enriched in layers 2/3 and low in layer 4 (Allen Brain Mouse Atlas). Consistent with this, 1-day monocular deprivation selectively remodels local excitatory inputs onto L2/3 PVs while sparing L4-derived inputs (Severin et al., 2021). Conversely, acute NRG1 potentiates L4 → L2/3 excitation onto PVs while leaving local inputs largely unaffected (Severin et al., 2021), supporting the idea that distinct molecular programs tune distinct afferent streams. Whether analogous context-dependence governs the recruitment of PVs in aging circuits remains to be determined.

Beyond development, the PV-recruitment model reframes age-related changes in inhibition. Here, an important question is not only “Do PVs fail?”, but also “How is PV recruitment retuned?”. Growing evidence suggests that successful cognitive aging reflects adaptive remodeling rather than simple resistance to change, and one influential hypothesis is that compensatory strengthening of inhibition supports preserved performance (Haberman et al., 2013; Branch et al., 2019). Notably, in behaviorally characterized aged rats performing an MTL-dependent task, aged unimpaired animals show higher NPTX2 expression in the dentate gyrus (Haberman et al., 2025) and stronger excitatory input onto dentate gyrus PVs (Severin et al., 2025). In both measures, aged unimpaired animals exceed aged impaired and young animals. These findings suggest that compensatory PV recruitment in aged unimpaired animals is mediated, at least in part, by NPTX2. This process is both an attractive therapeutic lever and an endogenous strategy to offset age-related circuit changes. How this compensation is implemented mechanistically—and whether it involves graded tuning, discrete transitions, or both—has further implications for how we conceptualize PV recruitment plasticity.

One possibility is that PV recruitment may follow a plasticity sequence that gates information flow digitally rather than in a graded manner. Activity could first induce LTD at excitatory synapses onto PVs (Huang et al., 2013; Chou et al., 2025), gradually reducing recruitment. Then, under appropriate conditions, this weakening could become all-or-none. During ODP, such disconnection appears structural—consistent with synapse elimination following monocular deprivation (Severin et al., 2021). Thus, PN → PV plasticity may operate not only as a dimmer but also as a switch, connecting or disconnecting inputs to determine whether specific excitatory pathways recruit PV inhibition at all.

Under less acute conditions, however, the progression may halt earlier: functional silencing without elimination. This would produce functional disconnection while preserving the structural substrate for reactivation. Such a mechanism could explain the increased PV recruitment in successful aging, where aged unimpaired animals show connectivity exceeding young animals (Severin et al., 2025; Haberman et al., 2025). Specifically, successful aging may involve expanding the pool of silent synapses and increasing their activation, with NPTX2 playing a central role. Whether silent synapses serve as an intermediate state—and whether their reactivation underlies successful aging—remains to be tested.

Several key directions emerge. First, we need to identify the natural conditions that engage PV recruitment plasticity, including NPTX2. Which patterns of experience, behavioral state, neuromodulation, or learning rules preferentially drive LTD-like weakening versus all-or-none disconnection? And how do these regimes evolve over development? Importantly, direct evidence for NPTX2-dependent PV recruitment plasticity in humans remains limited—such data would require invasive measures—and whether the mechanisms described here generalize beyond cortical and hippocampal structures awaits further study. Second, mapping how different recruitment mechanisms are deployed across circuits may reveal the ontogenetic origins of later-life compensation—whether age-related strengthening of PV recruitment reactivates developmental programs or reflects a distinct, late-emerging protective mode. Finally, recruiting PVs through their excitatory inputs is likely one strategy used to gate plasticity during development and compensate for declining inhibition in successful aging. It may also provide a promising therapeutic route to curb hyperexcitability in neurodegenerative disease.

## Data Availability

The original contributions presented in the study are included in the article/supplementary material, further inquiries can be directed to the corresponding author.

## Footnotes

1 We use MTL here to refer to the hippocampal–parahippocampal system, following convention in rodent memory studies.

2 Elevated activity observed at the network or regional level, such as increased neuronal firing rates or elevated BOLD signal, which can arise from underlying hyperexcitability.

3 An increased propensity of neurons or circuits to fire, due to altered intrinsic properties or reduced inhibitory restraint, which can manifest as network-level hyperactivity.
